# Ceftriaxone resistance among patients at GAMBY teaching general hospital

**DOI:** 10.1038/s41598-022-16132-3

**Published:** 2022-07-14

**Authors:** Litegebew Yitayeh Gelaw, Aschalew Afework Bitew, Eneyew Mebratu Gashey, Misrak Neway Ademe

**Affiliations:** 1 Public Health Department, Quality Officer, GAMBY Teaching General Hospital, Bahir Dar, Ethiopia; 2 Public Health Department, Research and Development Head, GAMBY Medical and Business College, Bahir Dar, Ethiopia; 3 Present Address: General Surgeon, GAMBY Teaching General Hospital, Bahir Dar, Ethiopia; 4 Microbiologist, GAMBY Teaching General Hospital, Bahir Dar, Ethiopia

**Keywords:** Microbiology, Health care

## Abstract

Ceftriaxone is a broad spectrum of widely used antibiotics as it is highly effective against Gram-negative and Gram-positive isolates. Research on Ceftriaxone resistance helps to know its current status. Hence, we aimed to identify the prevalence of Ceftriaxone resistance on bacteria isolated from clinical specimens among patients at GAMBY Teaching General Hospital Bahir Dar, Ethiopia. Hospital-based retrospective cross-sectional study was conducted at GAMBY teaching General Hospital from November 01, 2015, to December 30, 2020, on 402 clinical specimens. Ceftriaxone susceptibility tests were carried out using the Kirby-Bauer disc diffusion method. Descriptive statistics and chi-square tests were applied for the analysis. Escherichia coli 114 (28.4%), S. aureus 90 (22.4%), S. saprophyticus 42 (10.4%), and Klebsiella spp 42 (10.4%) were the predominant identified bacteria. The overall resistance of ceftriaxone was 230 (57.2%). Sex and type of the clinical specimens had significantly associated with its resistance whereas age was not associated with ceftriaxone resistance. Ceftriaxone resistance has been steadily increasing. Ceftriaxone resistance was high. Sex and type of the clinical specimens had significantly associated with its resistance. Prevention mechanisms to ceftriaxone resistance should be strictly implemented. The right drugs should be selected based on susceptibility patterns.

## Introduction

Antimicrobial resistance is one of the top global public health threat most commonly occurred due to bacteria^1^. Increasing antimicrobial resistance in bacteria that are important pathogens of humans and spread of resistance from the closed environment of hospitals into open communities is a threatening to public health^2^. Ceftriaxone is expected to be effective in a broad spectrum of gram positive or gram negative bacteria^3^.

In developing countries, we face particularities that go from antibiotic self-prescription to poor sanitary conditions, even at hospitals, that foster the threat of particular multi-resistant pathogens that are not common in developed countries and against which no new antibiotics are being investigated. In addition to the local consequences of these peculiarities upon resistance trends, it is important to realize that these can easily cross borders in this era of globalization^4^.

About 90% of deaths due to infections worldwide are caused by antibiotics resistance bacteria. Of these deaths, 45% are in low-income countries and 700,000 people die of resistance infection every year^5^.

Ceftriaxone is the third-generation Cephalosporin that is expected with excellent activity of many gram-negative and most gram-positive bacteria with its efficacy and safety in patients with respiratory tract, urinary tract, soft tissue, bone and joint infections, bacterial meningitis and gonorrhea^6^. It has long half-life which has resulted in a recommended once daily administration schedule of either intravenously or intramuscularly^7^.

A study done in Addis Ababa indicated that mortality is fivefold increased among patients with positive blood culture results. For this group of patients mortality is significantly associated with antimicrobial resistance. All 11 patients with Enterobacteriaceae resistant to third generation cephalosporins died. Eighty-nine patients had pancytopenia grade 3–4. Antimicrobial resistance that concerned gram-negative enteric bacteria, regardless of species, was characterized by co-resistance between third generation cephalosporins, gentamicin, chloramphenicol, and co-trimoxazole^8^.

Infection with antibiotic-resistant bacteria has been associated not only with increased morbidity, mortality but also costs of health care^9^. It also leads to additional costs, lengths of stay (LOS) due to an unsuitable or suboptimal therapy^10^ which reduces the chances of controlling complications in the most vulnerable patients^11^.

In low income countries, physicians rely on empirical treatments through broad spectrum antibiotics like ceftriaxone. Unfortunately, antimicrobial resistance is increasing due to over utilization of similar drugs repeatedly. Therefore periodic evaluation of drug resistance is mandatory to feed timely information on drug resistance specifically for broad spectrum drugs like ceftriaxone. Yes, there are already many reports of more broad studies from other countries. But continual supply of information especially on antibiotic resistance is very useful to give updated information on drug sensitivity. Also the strains of bacteria may be different in different areas which differ in ceftriaxone resistance. As prevalence of ceftriaxone prescription is high, up-to-date information related to ceftriaxone is very useful. So our study supports and helps for health care provider.Therefore, the aim of this study is to determine the prevalence of Ceftriaxone resistance in patients at GAMBY Teaching General Hospital Bahir Dar, Ethiopia.

## Methods and Materials

### Study area and setting

The study was conducted at GAMBY Teaching General Hospital (GTGH), private Hospital which is located in the Bahir Dar city. The hospital is offering medical, Gynecological, pediatrics and surgical treatment to about 100,000 patients in the outpatient department and 19,000 patients in the inpatient department every year. Patients of GAMBY Teaching General Hospital come from different areas either from urban or rural, from different region of the country. It also provides services for referral cases from governmental health centers especially for rural residences as the hospital is equipped with specialized services like Neurology, neurosurgery, ophthalmology, dermatology, Hepatobiliary and psychiatry. In addition to this, large number of emergency conditions especially car accidents flooded to the hospital each year^12^. The hospital laboratory equipped with a range of tests including culture and antimicrobial susceptibility test. It established Laboratory quality management system seven years back with step wise laboratory improvement towards accreditation (SLIPTA) and also accredited with GeneXpert scope by ISO (International Standardization for Organization) 15,189:2012 ENAO (Ethiopian National Accreditation Office) in 2019.

### Study design and period

Retrospective cross sectional was applied to review culture and susceptibility tests recorded data from November 01, 2015 up to December 30, 2020.

### Sample size and sampling techniques

Cultured and susceptibility tests from clinical samples in the study period were included. Totally, 402 records were eligible for the analysis.

### Operational definitions

**Body Fluid:** it included fluids taken from **peritoneal cavity or pleural cavity.**

**Sensitivity or Resistance:** A zone of inhibition as sensitive or resistant was interpreted according to Clinical and Laboratory Standards Institute^13^.

### Specimen processing and microbial identification

The clinical specimens included in this study were urine, wound swab, blood, body fluids, throat, cerebrospinal fluids, ear discharge, and genital swab either from outpatients or inpatients. The clinical samples were collected by standard microbiological technique. Initially, depending on the source of specimens, each sample were platted onto MacConkey agar, Blood agar, Mannitol Salt agar, Xylose lysine deoxycholate agar or Chocolate agar and then incubated aerobically at 37 °C for 24 h^14^. Gram-positive cocci in cluster, both catalase and coagulase positivity, and characteristically yellow to golden colored colonies on blood agar coupled with mannitol fermentation on MSA (Mannitol Salt Agar) were applied to identify Staphylococcus aureus from other gram-positive cocci. The gram-negative bacilli, the coliforms, Proteus spp., and Yersinia enterocolitica were identified by standard microbiological algorisms such as grams stain (gram-negative bipolarly stained bacilli for Yersinia spp) colonial growth characteristics and appearance on enriched and selective media^15^. Biochemical tests such as fermentation of lactose, glucose, and sucrose with and without H_2_S production using TSI/KIA (Triple Sugar Agar, Kliegler’s Iron Agar); lysine decarboxylation (LDC); indole and citrate utilization (MIS); methyl red (MR), Voges-Proskauer (VP); and pyrrolidonyl aminopeptidase (PYR) were used to identify the bacteria^13^. Thus, the identified bacteria from the clinical samples were *E. coli, S. aureus, Klebsella spp, K. pneumoniae, Citrobacter, S. pyogen, S. saprophyticus K. rihinous, S. epidermidis, S. typhi, and P. aeruginosa.*

### Antimicrobial susceptibility testing

Antimicrobial susceptibility testing was done using disk diffusion technique according to Kirby–Bauer method using S. aureus ATCC 25,923 and as quality control strains^16^ on the third-generation cephalosporin of ceftriaxone (30 μg). Accordingly, at least three to five well-isolated colonies of the same morphological type were selected from an agar plate culture and transferred into Muller Hinton broth and incubated at 37 °C for 24 h. The turbidity of the suspension was adjusted with sterile saline to obtain turbidity optically comparable to that of the 0.5 McFarland standards^17^. Then, the swab was streaked over the entire surface of the freshly prepared Mueller Hinton agar plate^18^. The antimicrobial disks were applied to the plates within 15 min after inoculation. The plates were then incubated at 37 °C for 24 h^19^. A zone of inhibition was measured and the results were interpreted as sensitive or resistant based on resistance data interpreted according to Clinical and Laboratory Standards Institute^13^.

### Quality control

The reliability of the study findings was guaranteed by implementing quality control measures throughout the whole process of the laboratory work. Staining reagents, culture Media, and antibiotic discs were checked for their normal shelf life before use. All culture plates and antibiotic discs were stored at recommended refrigeration temperature after being prepared and sterilized by autoclaving at 121 °C for 15 min. The standard reference bacterial strains were tested as a positive control on the biochemical tests and agar plates with antibiotic discs. Proper sample collection and handling were done by experienced microbiologist.

### Data analysis

Data were edited, cleaned, entered, and analyzed using Statistical Package for Social Science (SPSS) version 23. Frequency and percentage were used to describe socio-demographic characteristics of the patients. Bar graphs and tables were used to describe the results of the study. Chi-square (X^2^) test was calculated to compare the proportion of bacterial resistance with patients’ age, sex, and specimen type. P-value of < 0.05 was taken as cut point for statistically significant difference.

### Ethical approval and consent to participate

This study was conducted in accordance with the Declaration of Helsinki. Ethical approval was obtained from GAMBY Medical and Business College Research Review Committee. Written informed consent was taken from each respondent or from caregivers for children before they were requested to give samples by the attending physician. The procedures were carried out in accordance with relevant guidelines and regulations. The findings were reported to the attending physicians for the proper management of patients.

## Results

Urine, Wound, Blood, Body fluid, Throat, CSF (cerebrospinal fluid), Ear discharge, and Genital discharge clinical samples were collected from November 01, 2015 up to December 30, 2020. These samples were taken either from outpatient or inpatients. All these samples were tested for ceftriaxone resistance. Of the patients involved in the study, 276 (68.7%) were females. Approximately thirty percent, 119 (29.6%) were found in the age range 30 to 44 [see Table 1].

**Table 1:** Socio-demographic characteristics of sample source patients for ceftriaxone resistance.

Variables	Category	Frequency	Percent
Sex	Female	276	68.7
	Male	126	31.3
Age	0–14	95	23.6
	15–29	94	23.4
	30–44	119	29.6
	45–59	17	4.2
	> 60	77	19.2

**Figure 1:**
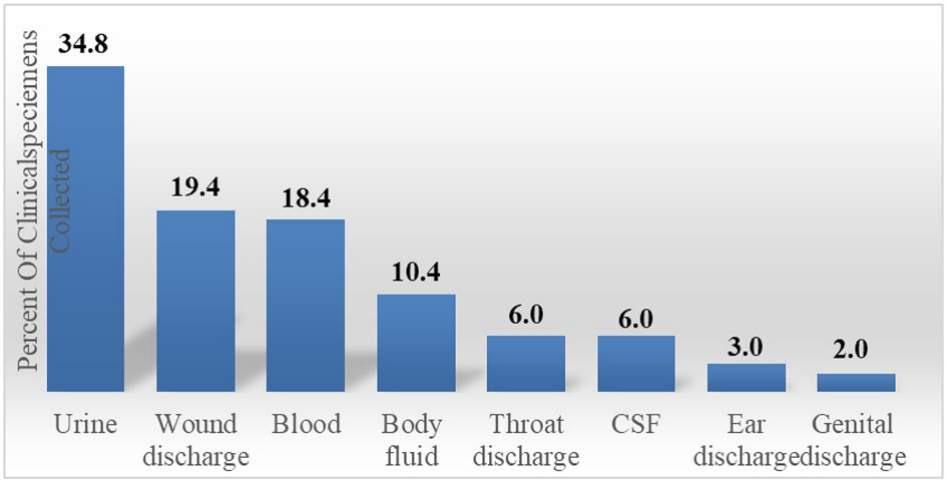
Showed the predominate clinical samples taken for drug susceptibility tests.

**Figure 2:**
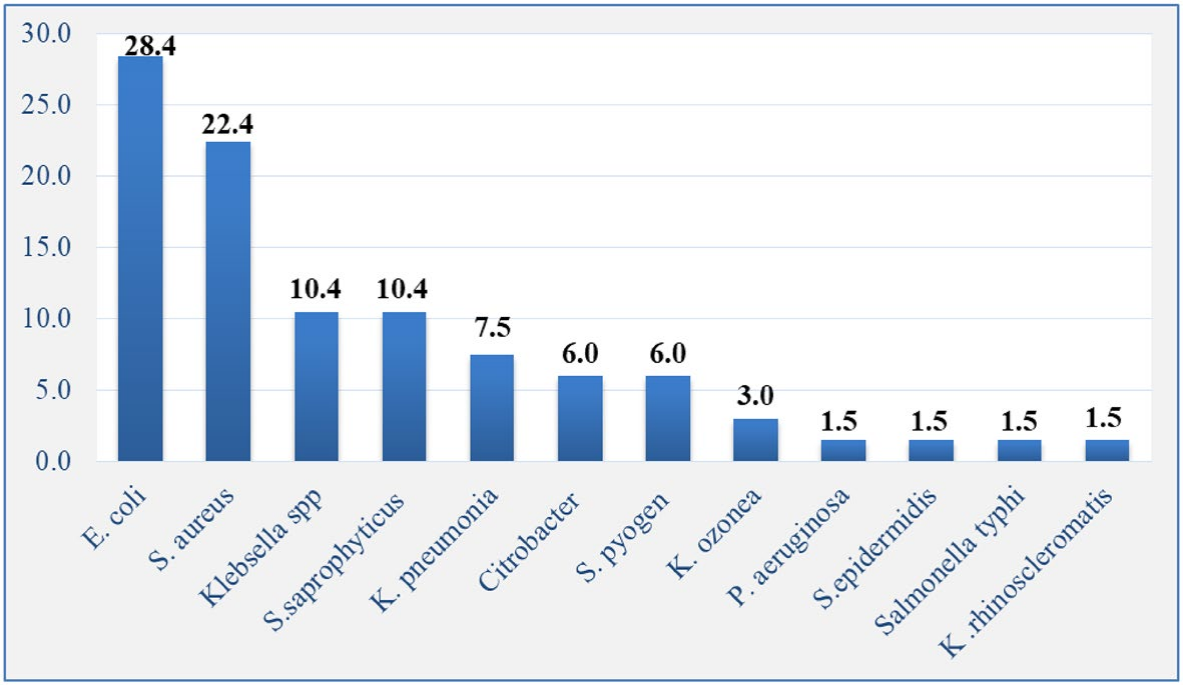
Showed the isolated bacteria from the clinical samples.

Of the collected clinical samples, the predominant were urine 140 (34.8%), wound 78 (19.4%), and Blood 74 (18.4%) processed respectively [see Fig. 1]. Totally, 12 types of bacteria were identified. Of these E. coli 114 (28.4%), *S. aureus* 90 (22.4%), *S. saprophyticus* 42 (10.4%), and *Klebsiella* spp 42 (10.4%) were the predominate bacteria respectively. The least bacteria isolated were *S. epideremides,* S. typhi, *P. aruginosa,* and k. rhinoscleromatis each accounted 6 (1.5%). Of 12 types of isolates, 8 (66.7%) were gram negative isolates [see Fig. 2].

The most commonly isolated bacterium in the urine was E. coli whereas *S. saprophyticus* was the predominated in the blood followed by *S.aureus* and *Klebiesella SPP.* In the body fluid, the predominated bacterium was *S.aureus* whereas *S.aureus* and *Klebiesella* SPP were the predominated bacteria in the wound discharge. The only bacterium identified in the genital discharge was *K. pneumoniae* whereas as the predominated bacterium in the throat swab was *S.pyogenes. S.aureus* and *S.pyogenes* were found equally in the ear discharge. *Klebsiella spps* was the predominated in the cerebrospinal fluid followed by *S.pyogenes and Kozaenae* [see Table 2].

The overall ceftriaxone resistance in isolated bacteria was 230 (57.2%). Majority of bacterial resistance was greater than fifty percent. The highest proportion of ceftriaxone resistance observed were *Klebsiella spp*, 36 (85.7%); *P. aeruginosa,* 5 (83.3%); *Staphylococcus saprophyticus,* 28 (66.7%); *Staphylococcus aureus,* 54 (60%) and *K. pneumoniae,* 18 (60%). Of the total resistance identified, higher number was recorded in gram-negative, 133 (58%) than gram positive which was 97(42%). [See Table 3].

**Table 2:** Cross tabulation of the clinical samples analyzed with the type of bacteria isolated.

Isolates	Urine	Blood	Wound discharge	Body fluid	Genital Discharge	Throat discharge	Ear discharge	CSF	Total
*E. coli*	90	0	18	6	0	0	0	0	114
*S. aureus*	12	12	42	18	0	0	6	0	90
*S. saprophyticus*	6	24	0	0	0	6	0	6	42
*Citrobacter *spp	12	6	6	0	0	0	0	0	24
*Klebisella *spp	0	12	0	18	0	0	0	12	42
*S. pyogenes*	0	0	0	0	0	18	6	0	24
*K. pneumoniae*	14	8	0	0	8	0	0	0	30
*K. ozaenae*	6	0	0	0	0	0	0	6	12
*S. epideremides*	0	6	0	0	0	0	0	0	6
*S. typhi*	0	0	6	0	0	0	0	0	6
*P. aruginosa*	0	0	6	0	0	0	0	0	6
*K. rhinoscleromatis*	0	6	0	0	0	0	0	0	6
Total	140	74	78	42	8	24	12	24	402

**Figure 3:**
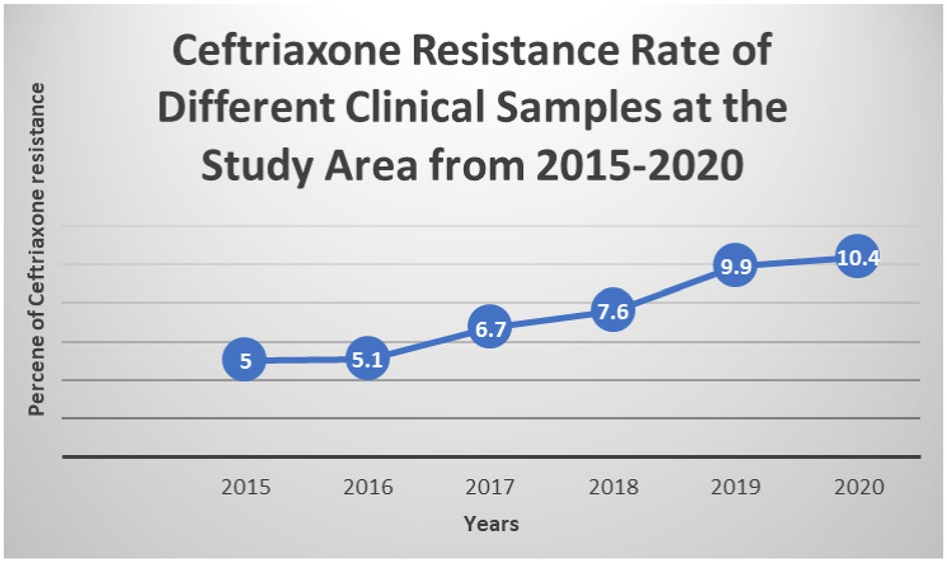
A trend line of Ceftriaxone resistance in 6 years period.

Ceftriaxone resistance was evaluated against sex, age, residence and type of clinical samples. As indicated below, in the Table 4 below, sex (X^2^ = 4.638; p-value = 0.031), residence (X^2^ = 4.24; p-value = 0.0395), and types of the clinical samples (X^2^ = 22.34; P-value = 0.005) had significantly associated with its resistance whereas age was not associated with ceftriaxone resistance (see Table 4). The line graph showed that there was alarmingly increasing resistance from 5 to 10.4 since 2015 up to 2020 [See Fig. 3].

**Table 3:** Overall bacterial resistance to ceftriaxone at GAMBY Teaching General Hospital.

Name of Bacteria	Total isolated	Resistance	Percentage
*E. coli*	114	54	47.4
*Staphylococcus aureus*	90	54	60.0
*Klebsiella spp*	42	36	85.7
*K. pneumoniae*	30	18	60.0
*Citrobacter*	24	12	50.0
*S. pyogenes*	24	12	50.0
*S. saprophyticus*	42	28	66.7
*P. aeruginosa*	6	5	83.3
*K. ozoneae*	12	4	33.3
*Staphylococcus epidermidis*	6	3	50.0
*Salmonella typhi*	6	2	33.3
*K. rhinoscleromatis*	6	2	33.3

**Table 4:** Association of resistance with some sociodemographic characteristics.

Variables	Category	Ceftriaxone sensitive	Ceftriaxone resistance	X^2^	P-value
Sex	Female	128 (31.8)	148 (36.8)	4.638	0.031
	Male	44 (10.9)	82 (20.5)		
Age	0–14	38 (9.4)	57 (14.2)	0.77	0.90
	15–29	39 (9.7)	55 (13.7)		
	30–59	61 (15.2)	75 (18.6)		
	> 60	34 (8.5)	43 (10.7)		
Residence	Urban	81 (20.1)	121(30.1)	4.24	0.0395
	Rural	60 (14.9)	140 (34.9)		
Specimen collected	Urine	65 (16.2)	73 (18.2)	22.34	0.005
	Wound	27 (6.7)	49 (12.2)		
	Blood and body fluid	55 (13.6)	83 (20.6)		
	Throat	16 (4.0)	8 (2.0)		
	Ear discharge	2 (0.5)	14 (3.5)		
	Genital discharge	8 (2.0)	2 (0.5)

## Discussion

Bacterial resistance is emerging rapidly and spread alarmingly. It is also significantly increasing as a challenge for physicians and caregivers worldwide. The overall resistance of ceftriaxone in isolated bacteria was 57.2% which was very high. This may be due to the fact that there is a malfunction activity relative to drug utilization which is supported by studies done in different part of the country as stated below: Large proportions (60%) were prescribed antibiotics and the most commonly prescribed single antibiotic was Ceftriaxone (21.7%), while ceftriaxone plus azithromycin was the most common combination (50.7%). The extent of non-adherence to the national guideline for the use of antibiotics was 36.4%^20^. Likewise, inappropriate use of antibiotics was 30.9% and inappropriate antibiotic intake, self-medication and family member medication were the common errors^21^.

The other possible justification for the large ceftriaxone resistance findings in our study was due to the fact that GAMBY Teaching General Hospital is used as referral hospital especially for rural populations either from governmental health center or primary hospital or private clinics.

In this study, more than half of the isolated bacteria were resistant to ceftriaxone and more resistance was observed in females 276 (68.7%) than males 126 (31.3%)***.*** This result was consistent with the study done in Dessie^22^.

There were great differences in the total resistance among different studies. This might be due to the difference in the study population, geographical variation, changes during sexual maturation, pregnancy, and genetic difference. The highest prevalence of microbial isolates was observed in the age groups of 15–44, (52.9%). This study was similar slightly higher than Debre Markos Referral Hospital which reported 54.9%^23^.

Furthermore, most of the isolates in this study were gram negative (60%) which was also similar with the study findings in tertiary care hospital, southern Ethiopia (62.77%)^24^. The total identified bacteria from urine, wound and blood clinical specimens constituted over 56.07% and this was less far from findings a cross-sectional study at Debre Markos Referral Hospital, Ethiopia^23^.

The most frequently isolated bacteria in this study were Escherichia coli 114 (28.4%). This finding was higher than Jimma which reported 25.3%^25^ and other studies done in northeast Ethiopia (14.2%)^26^, Debre Markos, Ethiopia (13.8%)^23^. However, our findings of E.coli was lower than the study done in Southern Ethiopia which indicated 42.9%^24^ and the study done in Gondar Comprehensive Specialized Hospital as it indicated 36%. The second, third and the fourth most isolated bacteria were *S. aureus* (22.4%), *S. saprophyticus* (10.4%), *and Klebsiella spp* (10.4%) respectively.

Regarding to ceftriaxone resistance, *Klebsiella spp* was 85.7%. This was higher than the studies conducted in Gondar comprehensive specialized hospital, 48.3%^27^ and at Jimma University medical center, 53.3%^25^. *P. aeruginosa* ceftriaxone resistance in the current study was *83.3%.* This finding was less than the study in Tikur Anbessa specialized hospital, Addis Ababa, Ethiopia 89.5%^28^. The possible reason for this may be tikur anbessa is specialized teaching hospital, more complicated cases referred to it.

*Staphylococcus saprophyticus isolates were 66.7%. This is more than the study done in* Jimma comprehensive specialized hospital which reported 14.3%^25^ and tikur Ambesa specialized hospital^28^. *K. pneumoniae was 60% which also higher than the study done* study Jimma comprehensive specialized hospital^25^*.* In this study the prevalence of ceftriaxone resistance in Staphylococcus aureus isolates was 60% which was higher than the study done in Gondar Referral Hospital, Northwest Ethiopia which reported 2*0.5% *^27^*.*

## Conclusion

Ceftriaxone resistance was high in the study. In addition, resistance to ceftriaxone was steadily increasing from 2015 to 2020. Sex, residence, and type of the clinical samples had significantly associated with its resistance. Prevention mechanisms to drug resistance should be strictly implemented to reduce the rate of ceftriaxone resistance. The right antibiotics should be selected based on susceptibility data.

## Data Availability

All data generated or analyzed during this study are included in the manuscript. However, the raw SPSS data are available from the corresponding author on reasonable request.

## Abbreviations

DTC: Drug and suppliers therapeutic Committee
KIA: Kliegler’s Iron Agar
LDC: Lysine decarboxylation
LOS: Lengths of Stay
MIS: Iindole and citrate utilization
MSA: Mannitol Salt Agar
PYR: Pyrrolidonyl aminopeptidase
TSI: Triple Sugar Iron
VP: Voges–Proskauer
WHO: World Health Organization
X^2^: Chi-square
